# Platelet Indices and Platelet to Lymphocyte Ratio (PLR) as Markers for Predicting COVID-19 Infection Severity

**DOI:** 10.7759/cureus.28206

**Published:** 2022-08-20

**Authors:** Rahul Ravindra, Prakriti Ramamurthy, Shaikh Mohammed Aslam S, Ashwin Kulkarni, Suhail K, Pranav S Ramamurthy

**Affiliations:** 1 Internal Medicine, Ramaiah Medical College, Bengaluru, IND; 2 General Medicine, Ramaiah Medical College, Bengaluru, IND; 3 Internal Medicine, Ramaiah Medical College & Hospital, Bengaluru, IND

**Keywords:** covid-19 severity, platelet indices, platelet count, covid-19 infection, platelet lymphocyte ratio

## Abstract

Background

Novel severe acute respiratory syndrome coronavirus 2 (SARS-CoV-2) (or coronavirus disease 2019; COVID-19) has caused a large number of infections across the globe. Numerous markers are being used to predict the severity of infection. This study was undertaken to assess the utility of platelet count, mean platelet volume (MPV), platelet distribution width (PDW), and platelet lymphocyte ratio (PLR) as markers of severity and mortality among patients with COVID-19 infection.

Methodology

This is a retrospective study conducted in a tertiary care center in India from April 2021 to June 2021. Patients admitted with COVID-19 infection were included in the study. Based on the severity, patients were categorized into the mild and severe (moderate severity included) groups. Platelet count, MPV, PDW, and PLR done at admission were studied and correlated with the disease severity and mortality.

Statistics

The independent t-test was used to compare the variables. The receiver operating characteristic (ROC) curve was done to identify the cut-off value. Statistical analysis was performed using SPSS 18 software (SPSS Inc. Released 2009. PASW Statistics for Windows, Version 18.0. Chicago: SPSS Inc).

Results

One hundred patients admitted with COVID-19 infection were studied. 51 patients had a mild and 49 had a severe infection. The mean PLR was 141.40 among patients with mild illness and 252.6 with severe infection (P<0.001). The mean PLR among survivors was 104.4 (SD-23.56) and among nonsurvivors was 302.78 (SD-34.5) (P<0.001). There was no statistically significant difference between the two groups with respect to platelet count, MPV, and PDW.

Conclusion

PLR was found to be a reliable marker of severity and mortality among patients with COVID-19 illness.

## Introduction

Novel severe acute respiratory syndrome coronavirus 2 (or coronavirus disease 2019; COVID-19) has caused a large number of infections and mortalities worldwide. A dysregulated immune response and cytokine storm is the underlying mechanism that determines the adverse outcome [1]. Numerous inflammatory markers can predict severity and mortality among patients with COVID-19. Platelet count, mean platelet volume (MPV), platelet distribution width (PDW), and platelet lymphocyte ratio (PLR) have been studied as markers of severity and mortality among patients with COVID-19 [2]. PLR is a marker of inflammation, which is economical and available in most clinical settings. PLR is a ratio between the absolute platelet count and absolute lymphocyte count. PLR has been used as a marker of inflammation in cardiovascular diseases and autoimmune diseases [2,3]. Platelet count and its variations are associated with an increased risk of severe disease and mortality in patients with COVID-19 and are a clinical indicator of worsening illness during hospitalization [4]. High PLR has been demonstrated as a good marker of severity and patients having elevated PLR had a longer duration of hospitalization. In COVID-19, the decrease in lymphocyte count is more marked than the decrease in platelet count. This explains the increase in PLR in COVID-19 infection [5]. MPV and PDW can be used as auxiliary tests in predicting mortality among patients with COVID-19 in a few studies. There is a paucity of such studies done in India. Hence, this study was undertaken to assess the utility of platelet count, MPV, PDW, and PLR as early markers of severity among admitted patients having COVID-19 infection in a tertiary care center in South India.

## Materials and methods

This is a retrospective study conducted in a tertiary care hospital in South India. The study was done after obtaining approval from the Institutional Ethics Committee of Ramaiah Medical College (MSRMC/EC/SP-07/04-2021). All procedures were carried out as per the Declaration of Helsinki 2013. Consecutive patients being admitted to the hospital suffering from COVID-19 infection were included in the study. A total of 100 adult patients (aged more than 18 years) were included in the study. COVID-19 infection was confirmed using reverse transcriptase polymerase chain reaction (RT-PCR COVID-19) on a throat swab. Patients suffering from any hematological diseases like leukemia, lymphoma, myelodysplastic syndrome, or idiopathic thrombocytopenic purpura, patients on chemotherapy, and patients on antiplatelet drugs were excluded, as these can alter the platelet count and platelet indices. As per Indian Council of Medical Research guidelines, patients with mild COVID-19 illness were those having symptoms of upper respiratory tract infection and/or fever without shortness of breath, moderate COVID-19 illness includes patients with a respiratory rate more than 24 per minute or oxygen saturation less than 93% on ambient air, and severe illness are those patients whose saturation is less than 90% on ambient air or respiratory rate more than 30 per minute [6]. For the purpose of the study and analysis, these patients were categorized into two groups, mild illness and severe illness (which included patients with moderate and severe illness). The data regarding these patients were extracted from the files and electronic health records. Care was taken not to collect or disclose the patients’ personal information. The clinical profile and laboratory data were studied. Complete blood counts, platelet count, MPV, PDW, and PLR, which were done at the time of admission were collected. The high-resolution computed tomography (HRCT) of the chest of the patients was also studied. The HRCT was reported by a radiologist. The correlation of platelet count, PLR, MPV, and PDW at admission with the severity and mortality of the illness was studied. Hemoglobin estimation was done using sodium lauryl sulfate detection, total leucocyte count estimation by a Sysmex device (Sysmex Corporation, Kobe, Japan), peripheral smear examination, C-reactive protein (CRP), and D-dimer measurement was done by immune-turbidometry.

Sample size with justification

A study conducted by Qu et al. has observed that the mean platelet to lymphocyte ratio among non-severe patients was 262.27 +/- 97.78 and among severe was 627.27 +/- 523.64, which was found to be statistically significant [4]. The present study expected similar results with 80% power, 95% confidence level, and an effect size of 0.64. The study required 98 patients; 49 in each group.

Statistical methods

Descriptive statistics of platelet count, lymphocyte count, PLR, and platelet indices were analyzed in both groups and summarized in terms of mean with standard deviation. The independent t-test was used to compare mean platelet count, MPV, PDW, and PLR between the two groups. The receiver operator characteristic (ROC) curve was constructed to identify the cut-off value, and the area under the curve (AUC) and the sensitivity and specificity of hematological parameters in predicting severity and mortality were analyzed. Correlation between parameters was done using Pearson’s correlation coefficient. Statistical analysis was performed using SPSS 18 (SPSS Inc. Released 2009. PASW Statistics for Windows, Version 18.0. Chicago: SPSS Inc.).

## Results

**Figure 1:**
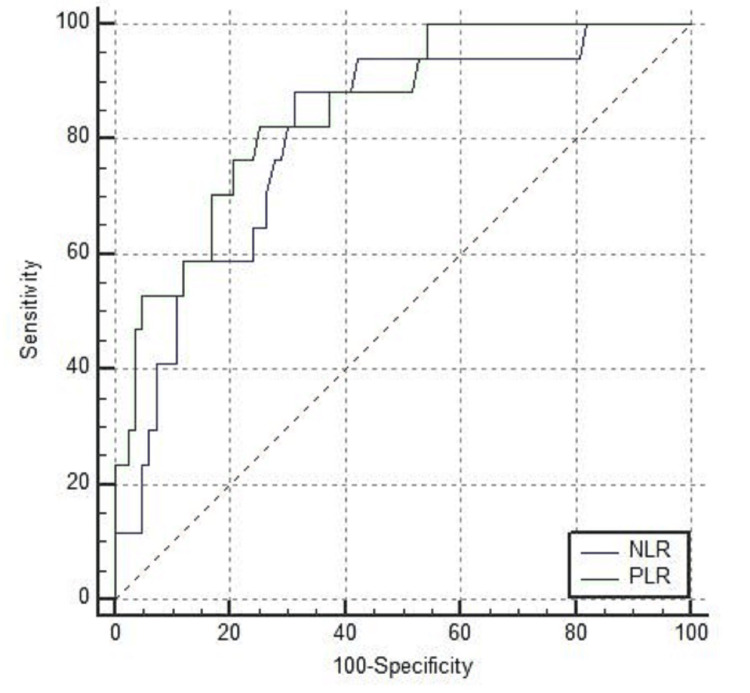
ROC curve comparison of NLR and PLR in predicting mortality ROC: receiver operating characteristic; NLR: neutrophil-lymphocyte ratio; PLR: platelet-lymphocyte ratio

**Figure 2:**
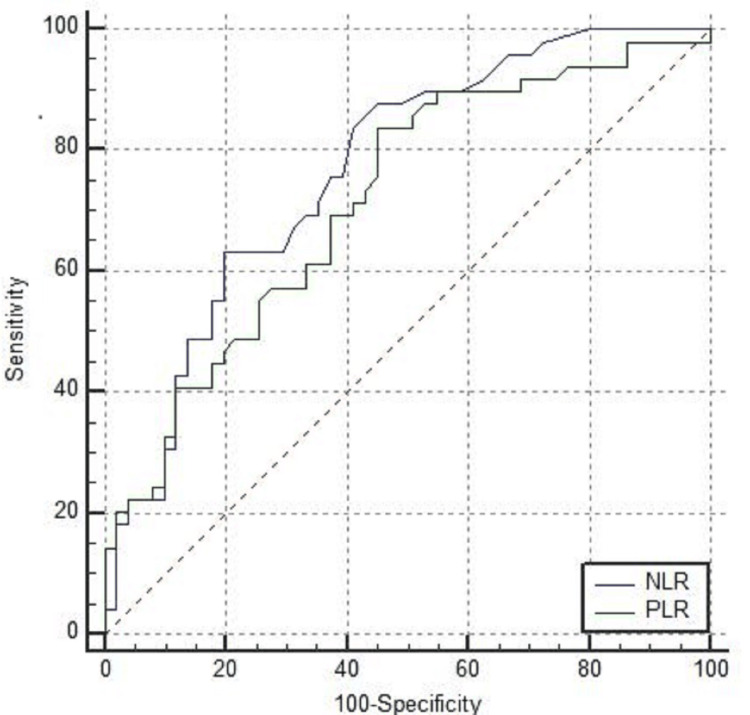
ROC curve comparison of NLR and PLR in predicting severity ROC: receiver operative characteristic; NLR: neutrophil-lymphocyte ratio; PLR: platelet-lymphocyte ratio

A total of 100 patients were included in the study. The baseline characteristics and symptoms are presented in Table 1. There were 51 patients in the mild illness category and 49 patients in the severe illness category. The mean age of patients in the mild category was 45.6 years (SD-16.1) and among severe cases was 55.6 years (SD-16.8). This difference was statistically significant (P=0.003). Fever was present in 24 (42.1%) patients with mild illness and in 33 (67.3%) with severe illness (p=0.04). Cough was present in 15 (31.3%) patients with mild illness and in 43 (87.7%) with severe illness (p<0.001). Breathlessness was present in one patient in the mild category and in 44 (89.7%) in the severe category (p<0.001). The mean platelet count among mild cases was 2.15 lakhs per milliliter (SD-93,221) and among severe cases, it was 2.06 lakhs per milliliter (SD-82,411) (P=0.5). MPV among mild cases was 8.7 (SD-1.1) femtoliter and among severe cases, it was 9.1 femtoliter (SD-1.5) (P=0.55). PDW was 17.11 (SD-0.73) among patients with mild illness and 16.47 (SD-2.16) among severe illness (P=0.06). PLR was 141.4 (SD-82.9) among patients with mild illness and 252.6 (SD-198.8) in patients with severe infections. This difference was statistically significant (P<0.001). NLR was 3.76 (SD-4.5) among mild cases and 8.44 (SD-8.8) among severe cases (P<0.001) (Table 2). There was a positive correlation between NLR and PLR with a CT severity score and a Pearson coefficient of 0.558 and 0.749, respectively (P<0.001) (Table 3). ROC was constructed for predicting mortality for NLR and PLR. The AUC for PLR was 0.851 (95% CI was 0.766 to 0.914). The AUC for NLR was 0.809 (95% CI was 0.718 to 0.880). For PLR with a cut-off of more than 200, sensitivity was 82.35% and specificity was 74.7% (Figure 1, Tables 4-5). For predicting severity, the AUC for NLR was 0.767 (95% CI was 0.766 to 0.914). The AUC for PLR was 0.718 (95% CI was 0.619 to 0.804). For predicting severity, PLR with a cut-off of more than 113, sensitivity was 83.67% and sensitivity was 54.9% (Figure 2, Tables 6-7). The mean PLR among survivors was 104.4 (SD-23.5) and among nonsurvivors was 302.7 (SD-34.5) (P<0.001). The mean platelet count among survivors was 2.3 lakhs per milliliter (SD-54,665) and among nonsurvivors, it was 2.1 lakh per milliliter(SD-45.654) (P=0.064). The mean MPV was 7.8 (SD-2.1) among survivors and 8.2 (SD-3.12) among nonsurvivors (P=0.12). PDW was 15.453 (SD-2.13) among survivors and 16.553 (SD-3.12) among nonsurvivors (P=0.078) (Table 8).

**Table 1:** Comparison of various parameters according to the severity of COVID-19 illness PDW: platelet distribution width; MPV: mean platelet volume; PLR: platelet lymphocyte ratio; CRP C-reactive protein

Laboratory Parameter	Severity of COVID-19 illness				P-value
	Mild (n=51)		Severe (n=49)		
	Mean	SD	Mean	SD	
Hemoglobin	13.80	2.17	13.05	2.04	0.081
Total leukocyte count	7000.00	3016.95	8444.90	3968.00	0.053
Neutrophils	4651.61	2910.12	6728.24	3892.14	0.003
Lymphocytes	1627.61	723.91	1189.02	772.05	0.004
Platelets	215901.96	93221.30	206448.	82411.08	0.551
PDW	17.11	7.3	16.47	2.16	0.064
MPV	8.71	1.15	9.10	1.54	0.157
NLR	3.76	4.50	8.44	8.86	0.001
PLR	141.40	82.92	252.60	198.86	<0.001
CRP	3.63	4.39	13.61	12.44	<0.001
D-DIMER	0.83	0.41	1.39	1.61	0.020

**Table 2:** Baseline characteristics, symptoms, and comorbidities among patients with mild or severe COVID-19 illness

Characteristics	Mild (N=51)	Severe (N=49)	P-value
Age (years)	45.63 (SD-16.132)	55.67 (SD-16.813)	0.003
Male	42 (82.3%)	34 (69.38%)	0.12
Female	9 (17.6%)	15 (30.6%)	0.12
Fever	24 (42.1%)	33 (67.3%)	0.046
Cough	15 (31.3%)	43 (87.7%)	<0.001
Breathlessness	1 (3%)	44 (89.7%)	<0.001
Diarrhea	5 (9.8%)	12 (24.4%)	0.02
Diabetes mellitus	22 (43.1%)	31 (63.2%)	0.048
Hypertension	18 (35.2%)	28 (57.15%)	0.044

**Table 3:** Correlation of NLR and PLR with CT severity score NLR: neutrophil-lymphocyte ratio; PLR: platelet-lymphocyte ratio; CT: computed tomography

Laboratory parameter	Correlation with CT severity score	
NLR	Pearson correlation	0.558
	P-value	<0.001
PLR	Pearson correlation	0.749
	P-value	<0.001

**Table 4:** Area under the curve for NLR and PLR in predicting mortality NLR: neutrophil-lymphocyte ratio; PLR: platelet-lymphocyte ratio

Laboratory parameter	Area under the curve (AUC)	95% CI
PLR	0.851	0.766 to 0.914
NLR	0.809	0.718 to 0.880

**Table 5:** Optimal cut-off values of NLR and PLR for predicting mortality NLR: neutrophil-lymphocyte ratio; PLR: platelet-lymphocyte ratio, PPV: positive predictive value; NPV: negative predictive value

	Cut-off	Sensitivity	Specificity	PPV	NPV
NLR	>3.9	88.24%	68.67%	36.6%	96.6%
PLR	>200	82.35%	74.7%	40%	95.4%

**Table 6:** AUC for NLR and PLR in predicting severity NLR: neutrophil-lymphocyte ratio; PLR: platelet-lymphocyte ratio; AUC: area under the curve

	AUC	95% CI
NLR	0.767	0.672 to 0.846
PLR	0.718	0.619 to 0.804

**Table 7:** Optimal cut-off values of NLR and PLR for predicting severity NLR: neutrophil-lymphocyte ratio; PLR: platelet-lymphocyte ratio; PPV: positive predictive value; NPV: negative predictive value

	Cut-off	Sensitivity	Specificity	PPV	NPV
NLR	>3.9	63.27%	80.39%	75.6%	69.5%
PLR	>113	83.67%	54.9%	64.1%	77.8%

**Table 8:** Differences in mean NLR, PLR, and platelet indices among survivors and nonsurvivors in COVID-19 infection PDW: platelet distribution width; MPV: mean platelet volume, NLR: neutrophil-lymphocyte ratio; PLR: platelet-lymphocyte ratio, SD: standard deviation

	Survivors (n=88)	Nonsurvivors (n=12)	P-value
Mean platelet count	2.23 lakhs/dL (SD-54,665)	2.11 lakhs/dL (SD-45,654)	0.064
Mean PDW	15.4 SD (2.13)	16.5 SD (3.12)	0.078
Mean MPV	7.8 SD (2.1)	8.2 SD (2.3)	0.12
Mean PLR	104.4 SD (23.56)	302.7 SD (34.5)	<0.001
Mean NLR	2.7 SD (4.5)	9.8 SD (6.8)	<0.001

## Discussion

The above study was conducted to study the utility of platelet count, MPV, PDW, and PLR as markers of severity of COVID-19 illness. The results of the above study indicate that PLR at admission was higher among the patients having severe COVID-19 illness as compared to the patients having a mild illness. PLR and NLR values had a significant positive correlation with CT severity scores. It was also observed that PLR at admission was found to be significantly elevated among patients who succumbed to COVID-19 infection as compared to patients who survived. The above study did not find any statistical difference in platelet count, mean platelet volume, and PDW among mild and severe illness. 

The findings of the present study were similar to the study done by Simadibrata DM et al., which showed that the patients with severe COVID-19 illness had higher PLR levels on admission (SMD 0.68; 95%CI 0.43-0.93; I2 =58%) [7].

Similar findings were observed in a study conducted by Rong Qu et al. which showed that values of PLR done at the highest platelet count were an independent factor that inversely influenced the outcome in patients with severe COVID-19 illness [4].

In the present study, PLR and NLR had a positive correlation with CT severity scores, which was statistically significant. These findings were concordant with the study conducted by Man A et al., which demonstrated that both NLR and PLR had a positive correlation with the radiological severity of COVID-19 illness as per CT severity scores. In this study, NLR was 2.90 and PLR was 186, having a good specificity and positive correlation with the CT severity score (0.89, p = 0.001; 0.92, p<0.001, respectively) [8].

Similarly, the study conducted by Eslamijouybari M et al, demonstrated that the values of NLR and PLR were elevated in COVID-19 patients compared to the control group (P=0.001) [9].

A review article was published by Chan AS et al. regarding NLR and PLR in COVID-19, which included 20 studies with 3,508 patients. As per this study, 19 studies demonstrated that NLR values were significantly elevated in patients with severe COVID-19 illness and five studies showed that PLR values were significantly elevated in patients with severe COVID-19 illness as compared to non-severe COVID-19 illness. Elevated levels of NLR (SMD: 2.80, 95% CI: 2.12 - 3.48, P < 0.00001) and PLR (SMD: 1.82, 95% CI: 1.03 - 2.61, P < 0.00001)) were observed in patients with severe COVID-19 illness compared to non-severe COVID-19 illness [10].

In the present study, the AUC for predicting COVID-19 severity for PLR was 0.851, and for NLR, it was 0.809. A study conducted by Jain R et al. demonstrated that mean NLR and PLR were significantly elevated in severe COVID-19 illness patients (NLR=7.41; PLR=204) compared with non-severe patients (NLR=3.30; PLR=121). ROC curve analysis showed that the AUC for NLR was higher than for PLR. An AUC of 0.779, with a larger OR of 1.237 and a cut-off of 4.1 showed 69% sensitivity and 78% specificity in predicting severe disease. The cut-off for PLR was 115.3, which showed 79% sensitivity and 62% specificity in predicting severe disease [11].

In the present study, it was seen that patients having severe COVID infection had more comorbidities like hypertension and type-2 diabetes mellitus. These findings were similar to the study by Huang et al. This study included 415 laboratory-confirmed COVID-19 patients. Compared with non-severe patients, severe patients exhibited more comorbidities, including hypertension (48.28% vs. 19.43%, p<0.001) and diabetes (20.69% vs. 6.99%, p=0.009). NLR and PLR were significantly elevated in patients with severe disease (p<0.001) [12].

A study conducted by Bg S et al. demonstrated that PLR and NLR were increased in around 60% of the patients with COVID-19 illness and were significantly increased in the patients who did not survive (p=0.004) [13].

Sarkar et al. retrieved 32 studies of COVID‐19 patients for outcomes. It was seen that critically ill patients and nonsurvivors had higher PLR levels on admission in comparison to survivors and non‐severe patients [14].

As per the results of the study conducted by Giuseppe Lippi et al., patients having severe COVID infections have significantly low platelet count [5]. However, in the present study, it was seen that patients with severe COVID had slightly low platelet count as compared with patients having a mild illness. But the difference was not statistically significant.

As per the study conducted by Seyit M et al., the CRP (p = 0.0001), lactate dehydrogenase (LDH) (p=0.038), PLR (p=0.0001), and NLR (p=0.001) were significantly higher in patients with a positive SARS-CoV-2 PCR test result [15]. Similar findings were also noted in the current study.

 PLR is a ratio between the absolute platelet count and the absolute lymphocyte count. In COVID-19 illness, the decrease in lymphocyte count is more marked than the decrease in the platelet count. This explains the increase in PLR in COVID-19 infection. SARS-CoV-2 infection can induce pyroptosis in lymphocytes through NLRP3 inflammasome activation. This mechanism leads to lymphopenia of COVID-19 illness. Cytokine IL-6 utilizes lymphocytes and can further reduce lymphocyte counts. Thus, an increase in PLR can be associated with severe COVID-19 illness [16].

The limitation of the study was that the sample size was small. Long-term follow-up and prospective studies will be required to understand the utility of these markers in COVID-19 infection.

## Conclusions

This study demonstrates that PLR can be used as a reliable marker of severity and mortality among patients with COVID-19 infection. PLR has a positive correlation with CT severity score. PLR is easily available as an economical marker and can be utilized in resource-limited settings. MPV, PDW, and platelet count were not found to be good markers of the severity of COVID-19 infections.
